# Thyroid imaging reporting and data system with MRI morphological features for thyroid nodules: diagnostic performance and unnecessary biopsy rate

**DOI:** 10.1186/s40644-024-00721-8

**Published:** 2024-06-14

**Authors:** Tingting Zheng, Yuan Zhang, Hao Wang, Lang Tang, Xiaoli Xie, Qingyin Fu, Pu-Yeh Wu, Bin Song

**Affiliations:** 1 Department of Radiology, Minhang Hospital, Fudan University, No 170, Xinsong Road, Minhang District, Shanghai, 201199 China; 2 Department of Ultrasound, Minhang Hospital, Fudan University, No 170, Xinsong Road, Minhang District, Shanghai, 201199 China; 3 Department of Pathology, Minhang Hospital, Fudan University, No 170, Xinsong Road, Minhang District, Shanghai, 201199 China; 4 GE Healthcare, MR Research China, Beijing, China

**Keywords:** Magnetic resonance imaging (MRI), Thyroid nodule, Diagnostic performance, Risk stratification, American College of Radiology Thyroid Imaging Reporting and Data System (ACR-TIRADS)

## Abstract

**Background:**

To assess MRI-based morphological features in improving the American College of Radiology Thyroid Imaging Reporting and Data System (ACR-TIRADS) for categorizing thyroid nodules.

**Methods:**

A retrospective analysis was performed on 728 thyroid nodules (453 benign and 275 malignant) that postoperative pathology confirmed. Univariate and multivariate logistic regression analyses were used to find independent predictors of MRI morphological features in benign and malignant thyroid nodules. The improved method involved increasing the ACR-TIRADS level by one when there are independent predictors of MRI-based morphological features, whether individually or in combination, and conversely decreasing it by one. The study compared the performance of conventional ACR-TIRADS and different improved versions.

**Results:**

Among the various MRI morphological features analyzed, restricted diffusion and reversed halo sign were determined to be significant independent risk factors for malignant thyroid nodules (OR = 45.1, 95% CI = 23.2–87.5, *P* < 0.001; OR = 38.0, 95% CI = 20.4–70.7, *P* < 0.001) and were subsequently included in the final assessment of performance. The areas under the receiver operating characteristic curves (AUCs) for both the conventional and four improved ACR-TIRADSs were 0.887 (95% CI: 0.861–0.909), 0.945 (95% CI: 0.926–0.961), 0.947 (95% CI: 0.928–0.962), 0.945 (95% CI: 0.926–0.961) and 0.951 (95% CI: 0.932–0.965), respectively. The unnecessary biopsy rates for the conventional and four improved ACR-TIRADSs were 62.8%, 30.0%, 27.1%, 26.8% and 29.1%, respectively, while the malignant missed diagnosis rates were 1.1%, 2.8%, 3.7%, 5.4% and 1.2%.

**Conclusions:**

MRI morphological features with ACR-TIRADS has improved diagnostic performance and reduce unnecessary biopsy rate while maintaining a low malignant missed diagnosis rate.

**Supplementary Information:**

The online version contains supplementary material available at 10.1186/s40644-024-00721-8.

## Key points

• This study used MRI morphological features to improve College of Radiology Thyroid Imaging Reporting and Data System (ACR-TIRADS).

• The performance of the improved ACR-TIRADS is superior to that of the conventional ACR-TIRADS.

• Magnetic resonance morphology features may reduce unnecessary biopsy rate while maintaining a low missed diagnosis rate, thus avoiding unnecessary invasive diagnosis and treatment.

**Supplementary Information:**

The online version contains supplementary material available at 10.1186/s40644-024-00721-8.

## Introduction

Thyroid nodules are a prevalent issue in the endocrine system. The utilization of high-resolution ultrasound has greatly enhanced the ability to detect thyroid nodules and prevalence rates ranging from 19–68% in randomly selected individuals [1–3]. The majority of these nodules are benign, with only a small proportion having clinical significance and approximately 5–10% being confirmed as thyroid cancers [4]. The global prevalence of thyroid cancer continues to increase, currently ranking as the fifth most common form of cancer among American women [5, 6]. The assessment of the benign or malignant nature of thyroid nodules is of utmost importance in selecting the appropriate treatment decision-making, avoiding unnecessary biopsy rate, and improving the disease prognosis [4].

Ultrasonography presently serves as the primary imaging modality for distinguishing between benign and malignant thyroid nodules [7]. Thyroid Imaging Reporting and Data System (TI-RADS) is routinely utilized to effectively manage thyroid nodules by relying on ultrasound risk characteristics [8–10]. Among the various TI-RADSs, the American College of Radiology TI-RADS (ACR-TIRADS) [11] demonstrates superior diagnostic efficacy and a lower unnecessary biopsy rate. Through the examination of a substantial sample size of 37,585 thyroid nodules, Kim et al. [12] found that the ACR-TIRADS had high sensitivity (> 90%) for TI-RADS 4 and 5 nodules, with a specificity of only 49%. This low specificity resulted in a considerable number of benign nodules being misdiagnosed, leading to unnecessary biopsy. Therefore, it is imperative to optimize the ACR-TIRADS system to improve specificity and the unnecessary biopsy rate.

Magnetic resonance imaging (MRI) presents numerous advantages, including the ability to conduct imaging in arbitrary planes, the absence of ionizing radiation, exceptional soft tissue contrast, and the capacity to capture various qualitative and quantitative features [13, 14]. Diffusion-weighted imaging (DWI) is a functional imaging technique that provides molecular signatures about pathological conditions and underlying pathophysiological mechanisms by capturing the random Brownian motion of water molecules in tissues [15]. Furthermore, contrast-enhanced (CE) MRI is commonly adopted to assess the process of lesion enhancement and clearance of contrast material, playing a pivotal role in the identification of both benign and malignant tumors [16–18]. Multiple studies have provided evidence for the effectiveness of multiparametric MRI in diagnosing both benign and malignant thyroid nodules [19–23]. The use of MRI-based features to improve the ACR-TIRADS has not yet been thoroughly investigated in study, though. Therefore, this study aimed to improve the ACR-TIRADS by integrating the MRI-based morphological features, and investigate the efficacy of various improved methods in enhancing the diagnostic accuracy for distinguishing between benign and malignant thyroid nodules, as well as reducing the unnecessary biopsy rate.

## Materials and methods

### Patients and study design

The ethical committee of Minhang hospital, Fudan University approved this retrospective observational study that informed consent was abandoned, which was carried out in accordance with the guidelines specified in the Declaration of Helsinki.

Retrospective research was done on 931 thyroid nodules from 688 individuals who underwent surgical excision at our facility between January 2017 and December 2022. Inclusion criteria were as follows: (1) patients who underwent preoperative thyroid MRI; (2) patients with postoperative pathological confirmation as benign or malignant. Exclusion criteria were as follows: (1) presence of diffuse bilateral lesions with different pathological types; (2) poor image quality with severe artifacts; (3) patients who underwent FNA or partial thyroidectomy prior to MRI; (4) cases with unclear postoperative pathological findings; (5) incomplete imaging; (6) lesions smaller than 5 mm; (7) absence of preoperative thyroid US or insufficient US images for diagnosis. The surgical indications for thyroid nodules include those categorized as TI-RADS grade ≥ 4, indicating a high suspicion of thyroid cancer, as well as symptomatic benign thyroid tumors resulting from compression, hyper-functioning thyroid adenomas, or concomitant hyperthyroidism.

**Fig. 1:**
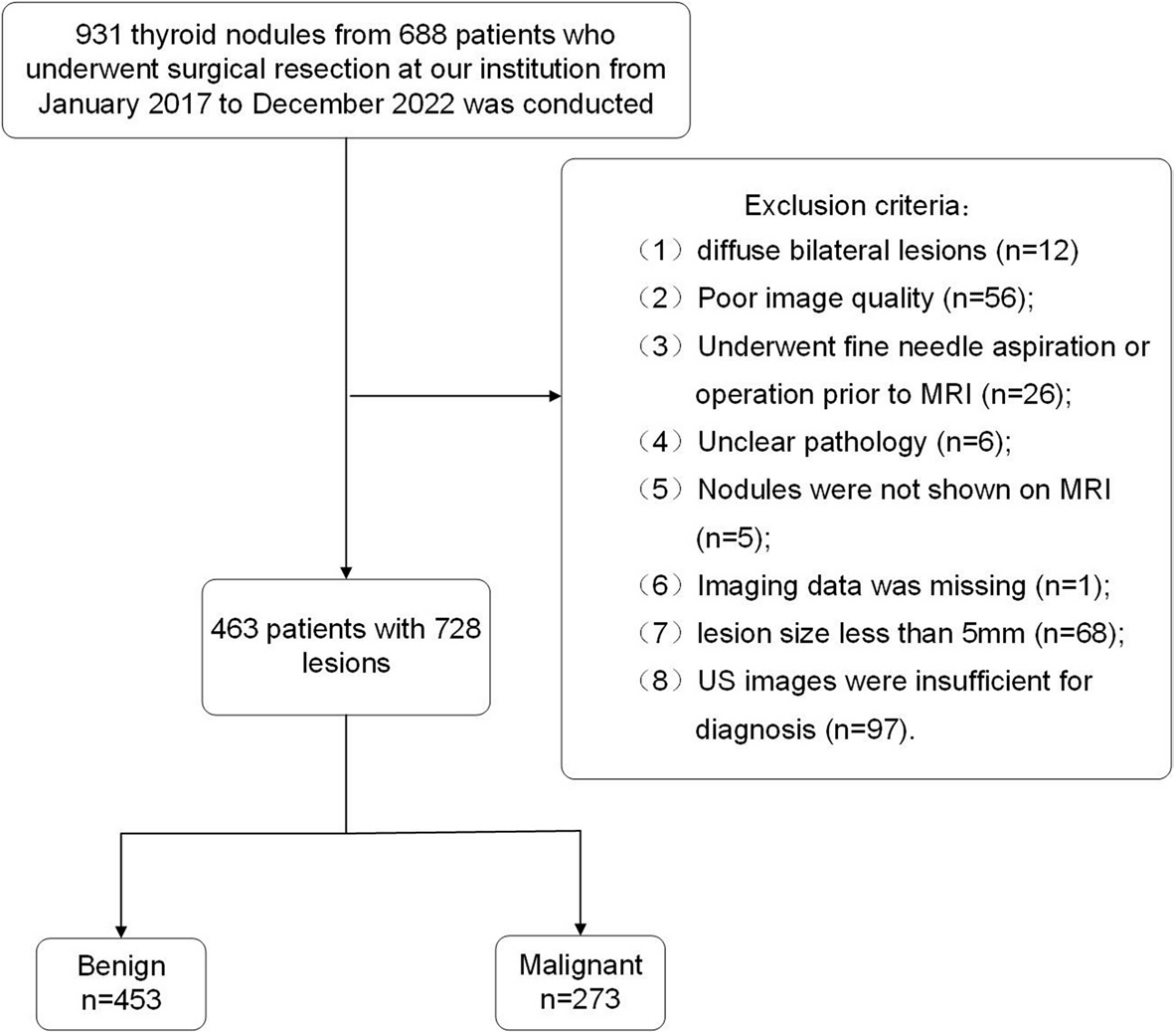
Flowchart of participant selection

Ultimately, the study comprised a total of 463 participants with 728 lesions, consisting of 453 benign lesions and 275 malignant lesions (Fig. 1).

### MRI acquisition

MRI examinations were performed on a 1.5 T MRI scanner (Excite HD; GE Healthcare, Milwaukee, WI, USA) equipped with an 8-channel phased-array thyroid coil (Chenguang Medical Technologies, Shanghai, China). The MRI protocols included: (1) coronal fat-suppressed T2-weighted imaging (T2WI) was acquired by a fast recovery fast spin echo (FRFSE) sequence; (2) axial fat-suppressed T2WI; (3) axial T1-weighted imaging (T1WI) was acquired by a FSE sequence; (4) DWI was acquired by a spin-echo echo-planar imaging (SE-EPI) sequence (b-values = 0 and 800 s/mm^2^); (5) multiphasic CE T1WI was acquired by a fast spoiled gradient echo (FSPGR) sequence at 30 s, 60 s, 120 s, 180 s, 240 s and 300 s after contrast injection. The comprehensive MRI acquisition parameters are shown in Supplementary Table 1. A 15 ml saline flush was administered immediately after the contrast agent (Magen Vixen; Bayer Pharmaceuticals, Berlin, Germany) was injected at a rate of 0.0306 mmol/kg. The total scan time was approximately 14 min.

### MRI morphological analysis

Two radiologists (with 6 and 10 years of experience in thyroid MRI), who were unaware of the pathology of lesions, independently evaluated the MRI images using Advantage Workstation 4.5 (GE Healthcare) and Picture Archiving and Communication System (PACS). In case where there was a disagreement between them, a consensus was reached through discussion. The characteristics of lesion were evaluated as follows: (1) restricted diffusion was defined as lesion with high signal on DWI and low signal on corresponding apparent diffusion coefficient (ADC) maps; (2) reversed halo sign was defined as lesion in the delayed phase of CE-T1WI exhibiting high signal intensity in the peripheral area compared to the central area and a blurred outline.

### ACR-TIRADS

Two experienced ultrasonography experts, each with over a decade of experience, conducted a retrospective analysis of the ultrasonography characteristics of thyroid nodules. These experts were unaware of the histopathological results during the analysis. Subsequently, they reached a consensus on the various ultrasonography features, encompassing composition, echogenicity, margin, shape, calcification, aspect ratio, extrathyroidal extension, and suspicious cervical lymph node. The ACR-TIRADS was used to categorize each thyroid nodule. Detailed ACR-TIRADS classification are shown in Supplementary Table 2 and 3.

### Improved ACR-TIRADS risk stratification systems

The development of four improved ACR-TIRADS risk stratification systems was facilitated by utilizing the presence or absence of the two MRI-based morphological features in the lesion. The four conditions were as follows: (1) solely exhibiting restricted diffusion; (2) solely displaying the reversed halo sign; (3) manifesting both restricted diffusion and the reversed halo sign; (4) presenting either restricted diffusion or reversed halo sign. Whenever any of the aforementioned four conditions are present in a nodule, the ACR-TIRADS level is upgraded by one level, and conversely, downgraded by one level (the original TI-RADS categorization of grade 2 is not downgraded, and the original TI-RADS categorization of grade 5 is not upgraded).

### Comparison of conventional and improved ACR-TIRADSs

The sensitivity, specificity, accuracy, positive predictive value (PPV), and negative predictive value (NPV) of conventional and different improved ACR-TIRADS were separately calculated.

The unnecessary biopsy rate and malignant missed diagnosis rate were compared between conventional and four improved ACR-TIRADSs. The unnecessary biopsy rate refers to the percentage of benign nodules among those recommended for biopsy according to the ACR-TIRADS guidelines. On the other hand, the malignant missed diagnosis rate represents the percentage of malignant nodules determined to be less than grade 4 according to different ACR-TIRADS system.

### Statistical analysis

All statistical analyses were performed using SPSS 26.0 (IBM Corp, Armonk, NY, USA) and MedCalc 19.2.1 (MedCalc Software bv, Ostend, Belgium) software packages. Continuous variables were presented as mean ± standard deviation (SD), while categorical variables were expressed as percentages. T-test and Chi-square test or Fisher’s exact test were employed for comparing continuous and categorical variables, respectively. Kappa concordance test was utilized to evaluate concordance between two radiologists. Receiver operating characteristic (ROC) analysis was conducted to assess the diagnostic performance of different systems, and the area under the ROC curve (AUC) was recorded. Delong test was adopted to compare the AUC values. All statistical tests were two-tailed, and *p*-values along with 95% confidence interval (CI) were reported. *P* values < 0.05 were considered statistically significant.

## Results

### Clinicopathological characteristics

The clinical baseline and pathological data of the patients are shown in Table 1. With the exception of gender, all variables exhibited significant differences in the distribution between benign and malignant nodules. A total of 463 patients (mean age, 51.33 ± 13.85 years) with 728 thyroid nodules were included in the study, and were classified as benign (*n* = 453) or malignant (*n* = 275) based on the pathological findings following surgical resection.

**Table 1:** Basic clinical information of the 463 patients with 728 thyroid nodules

Characteristics	Benign	Malignant	Total	*p* value
Age, mean ± SD, years	55.58 ± 12.12	44.34 ± 13.70	51.33 ± 13.85	< 0.001^*^
Gender				0.291
Male	114(25.2)	79(28.7)	193(26.5)	
Female	339(74.8)	196(71.3)	535(73.5)	
Number				< 0.001^*^
Unifocal	82(18.1)	142(51.6)	224(30.8)	
Multifocal	371(81.9	133(48.4)	504(69.2)	
Location				0.016^*^
Left lobe	217(47.9)	110(40.0)	327(44.9)	
Right lobe	206(45.5)	154(56.0)	360(49.5)	
Isthmus	30(6.6)	11(4.0)	41(5.6)	
Size				< 0.001^*^
0.5-1 cm	116(25.6)	124(45.1)	240(33.0)	
1-4 cm	264(58.3)	134(48.7)	398(54.7)	
≥ 4 cm	73(16.1)	17(6.2)	90(12.4)	
Hashimoto’s thyroiditis				< 0.001^*^
Absent	391(86.3)	208(75.6)	599(82.3)	
Present	62(13.7)	67(24.4)	129(17.7)	

Data are expressed as the number of nodules, with percentages in parentheses

*Abbreviations*: *SD* standard deviation

^*^p < 0.05

### MRI-based morphological features

**Fig. 2:**
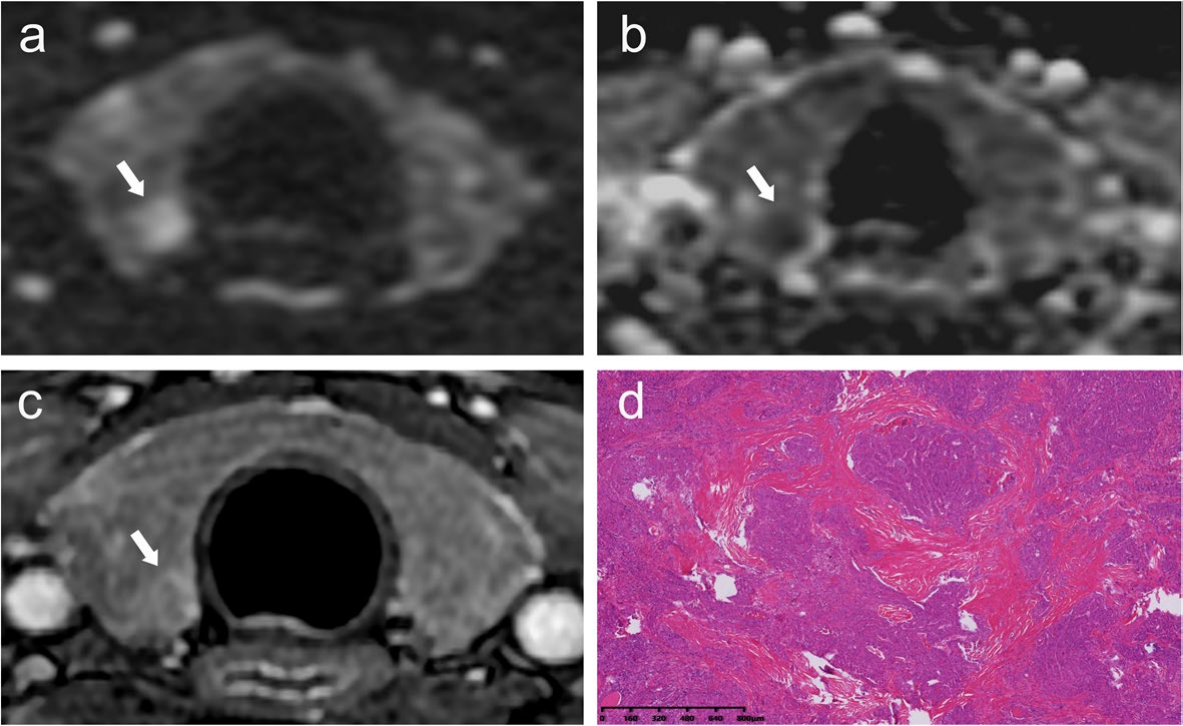
A 38-year-old female presented with papillary thyroid carcinoma in the right lobe. Axial diffusion-weighted imaging (DWI) (**a**) and apparent diffusion coefficient (ADC) map (**b**) demonstrate restricted diffusion of the lesion, indicated by high signal intensity on DWI and low signal intensity on ADC, as denoted by the white arrow. Delayed phase contrast-enhanced T1-weighted imaging (**c**) reveals central decreased enhancement of the nodule with relative hyperenhancement at the periphery, displaying a reversed halo sign. Histopathological examination using hematoxylin and eosin staining at low magnification (HE, × 1) (**d**), demonstrates densely distributed tumor cells within a fibrous stromal component

Table 2 displays the statistical analysis results of MRI-based morphological features. The presence of restricted diffusion (*P* < 0.001, OR = 45.1) and the reversed halo sign (*P* < 0.001, OR = 38.0), both indicating a good agreement (Kappa value, 0.914 and 0.818), were identified as independent predictors for malignant thyroid nodules. Representative images of two MRI morphological features with pathology are shown in Fig. 2.

**Table 2:** Univariate and multivariate analyses results of benign and malignant thyroid nodules

Characteristics	Benign (*n* = 453)	Malignant (*n* = 275)	*p*	Multivariate Analysis		Kappa
				OR (95%CI)	*p*	
Restricted diffusion			< 0.001^*^	45.1(23.2–87.5)	< 0.001^*^	0.914
Absent	426 (94.0)	49 (17.8)				
Present	27(6.0)	226 (82.2)				
Reversed halo sign			< 0.001^*^	38.0(20.4–70.7)	< 0.001^*^	0.818
Absent	434 (95.8)	57 (20.7)				
Present	19 (4.2)	218 (79.3)				

Data are expressed as the number of nodules, with percentages in parentheses

*Abbreviations*: *OR* odds ratio, *CI* confidence interval

^*^p < 0.05

### Diagnostic performance of conventional and improved ACR-TIRADSs

The grading results and malignancy rates of thyroid nodules based on the conventional and improved ACR-TIRADS are shown in Table 3. In the conventional ACR-TIRADS, there were a total of 259 thyroid nodules classified as TI-RADS 4, whereas in the four improved ACR-TIRADSs, the numbers of TI-RADS 4 were 59, 58, 74, and 43 for each respective system. Comparison of the upgraded and downgraded nodules in different improved methods is presented in Supplement Material Table 4.

**Table 3:** Classification results of thyroid nodules by the conventional and four improved ACR-TIRADSs

Methods	Classification	Benign	Malignant	Total	Rate of malignancy (%)	*p value*
ACR-TIRADS						< 0.001^*^
	TI-RADS 2	5	0	5	0.0	
	TI-RADS 3	253	8	261	3.0	
	TI-RADS 4	172	87	259	33.6	
	TI-RADS 5	23	180	203	88.7	
Restricted diffusion (A)						< 0.001^*^
	TI-RADS 2	253	3	256	1.2	
	TI-RADS 3	152	18	170	10.6	
	TI-RADS 4	26	33	59	55.9	
	TI-RADS 5	22	221	243	90.9	
Reversed halo sign (B)						< 0.001^*^
	TI-RADS 2	251	6	257	2.3	
	TI-RADS 3	164	21	185	11.4	
	TI-RADS 4	26	32	58	55.2	
	TI-RADS 5	12	216	228	94.7	
A + B						< 0.001^*^
	TI-RADS 2	258	7	265	2.6	
	TI-RADS 3	169	32	201	15.9	
	TI-RADS 4	23	51	74	68.9	
	TI-RADS 5	3	185	188	98.4	
A or B						< 0.001^*^
	TI-RADS 2	246	2	248	0.8	
	TI-RADS 3	147	7	154	4.5	
	TI-RADS 4	29	14	43	32.6	
	TI-RADS 5	31	252	283	89.0	

*Abbreviations*: *ACR* American Radiology, *TIRADS* Thyroid Imaging Reporting and Data System

^*^p < 0.05

**Fig. 3:**
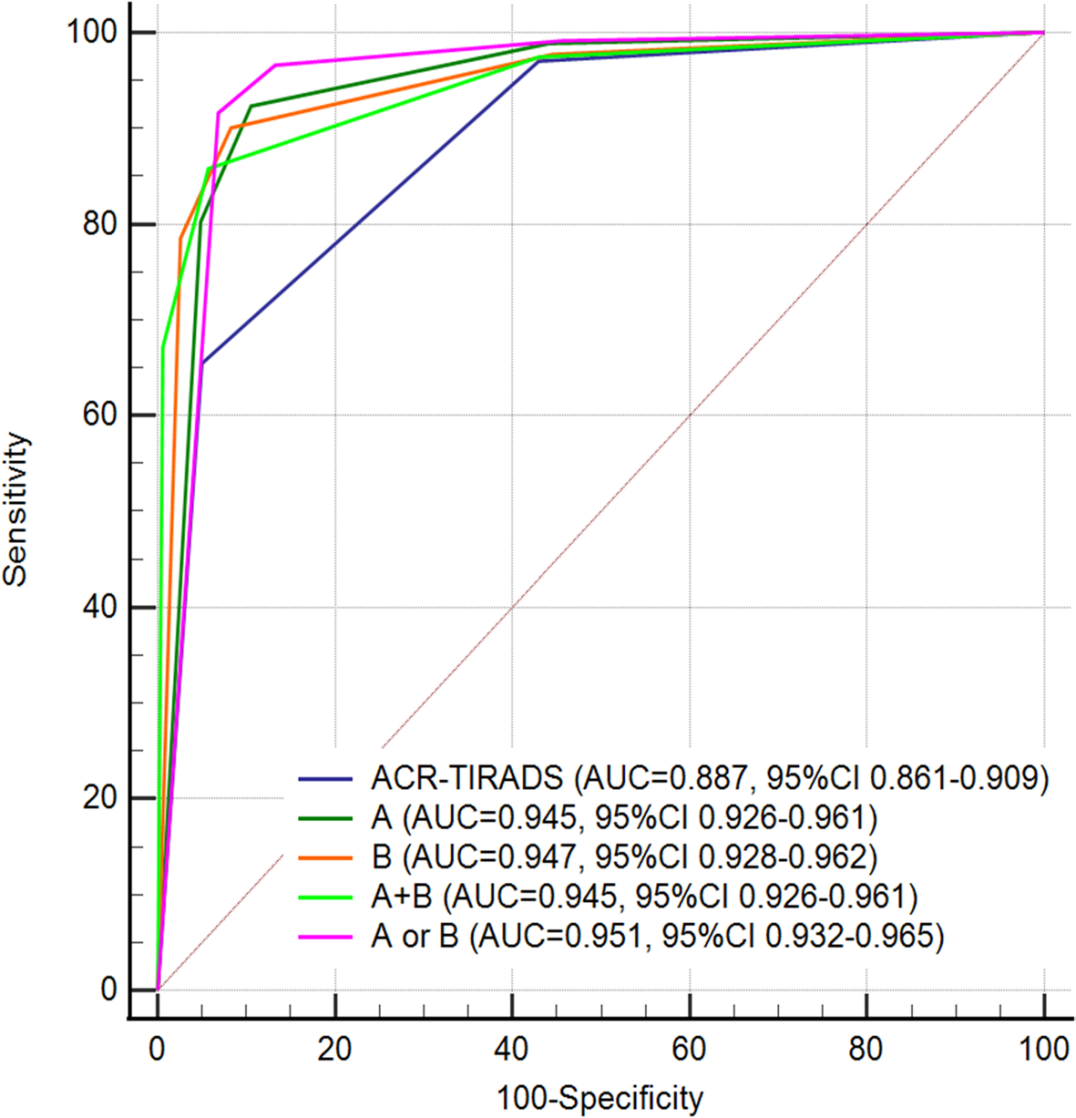
Receiver operator characteristic curves for conventional and different improved ACR-TIRADS. **A** Restricted diffusion; **B** Reversed halo sign

The ROC curves and AUCs of the conventional and four improved ACR-TIRADS are shown in Fig. 3 and Table 5. The AUCs of conventional and four improved ACR-TIRADS were 0.887 (95% CI: 0.861–0.909), 0.945 (95% CI: 0.926–0.961), 0.947 (95% CI: 0.928–0.962), 0.945 (95% CI: 0.926–0.961) and 0.951 (95% CI: 0.932–0.965) respectively, and between the differences were statistically significant (Delong test, ACR-TIRADS VS improved ACR-TIRADSs, all *p* < 0. 005).

**Fig. 4:**
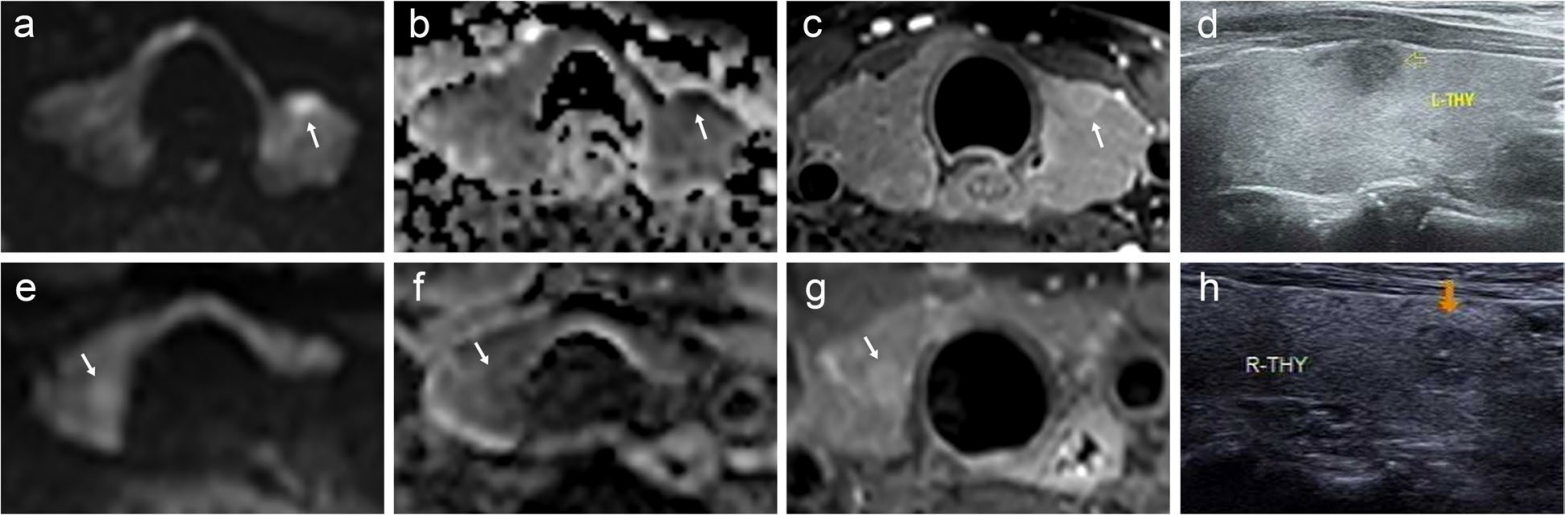
A 51-year-old female with papillary thyroid carcinoma in the left lobe (**a**, **b**, **c** and **d**). Axial diffusion-weighted imaging (DWI) (**a**) and apparent diffusion coefficient (ADC) map (**b**) show restricted diffusion of the lesion (white arrow). The delayed phase contrast-enhanced T1-weighted imaging (**c**) shows reversed halo sign (white arrow). The ultrasound image (**d**) displays a lesion initially classified as ACR-TIRADS grade TI-RADS 4, which has been upgraded to the improved ACR-TIRADS grade of TI-RADS 5, indicating the recommendation for fine needle aspiration biopsy. A 67-year-old female with adenomatous goiter in the right lobe (**e**, **f**, **g** and **h**). Axial DWI (**e**) and ADC (**f**) show absence of restricted diffusion (white arrow). The delayed phase contrast-enhanced T1-weighted imaging (**g**) shows a relatively homogeneous enhancement pattern (white arrow). The ultrasound image (H) of the lesion, initially classified as ACR-TIRADS grade TI-RADS 4 but subsequently revised to an improved ACR-TIRADS grade TI-RADS 3, obviating biopsy

The diagnostic performance of the conventional and four improved ACR-TIRADSs is provided in Table 4. Representative ultrasound and MRI images of thyroid nodules are shown in Fig. 4.

**Table 4:** The diagnostic performance of the conventional and four improved ACR-TIRADSs

Methods (cutoff)	Sensitivity	Specificity	Accuracy	PPV	NPV
ACR-TIRADS (≥ 4)	97.1	57.0	72.1	57.8	97.0
Restricted diffusion (A) (≥ 4)	92.4	89.4	90.5	84.1	95.1
Reversed halo sign (B) (≥ 4)	90.2	91.6	91.1	86.7	93.9
A + B (≥ 4)	85.8	94.3	91.1	90.1	91.6
A or B (≥ 4)	96.7	86.8	90.5	81.6	97.8
ACR-TIRADS (≥ 5)	64.6	94.7	83.8	88.2	81.4
Restricted diffusion (A) (≥ 5)	80.4	95.1	87.2	90.9	88.9
Reversed halo sign (B) (≥ 5)	78.5	97.4	90.3	94.7	88.2
A + B (≥ 5)	67.3	99.3	87.2	98.4	83.3
A or B (≥ 5)	92.6	91.6	93.1	94.0	94.8

Data are expressed as percentages

*Abbreviations*: *PPV* positive predictive value, *NPV* negative predictive value, *ACR* American College of Radiology, *TIRADS* Thyroid Imaging Reporting and Data System

Supplementary Table 5 provides the diagnostic performance of the MRI-based morphological features, conventional and four improved ACR-TIRADSs across three different groups based on thyroid nodule size. For nodules of 1 cm or smaller, the accuracy of ACR-TIRADS was 69.2% (166/240), while the four improved ACR-TIRADSs achieved higher accuracy of 88.4% (212/240), 88.4% (212/240), 88.4% (212/240) and 86.3% (207/240), respectively, surpassing that of the ACR-TIRADS. Among nodules ranging in size from 1 to 4 cm, the accuracy of ACR-TIRADS was 73.4% (292/398), while the four improved ACR-TIRADSs exhibited accuracy of 93.2% (371/398), 93.7% (373/398), 93.7% (373/398), and 93.2% (371/398), respectively. For nodules larger than 4 cm, the accuracy of ACR-TIRADS was 74.4% (67/90), while the four improved ACR-TIRADSs were 90.0% (81/90), 86.7% (78/90), 86.7% (78/90), and 90.0% (81/90), respectively. Supplementary Table 6 provides a detailed overview of the diagnostic performance of the conventional ACR-TIRADS and four improved ACR-TIRADSs in various pathological types.

### The rate of unnecessary biopsy rate and malignant missed diagnosis

The unnecessary biopsy rate and malignant missed diagnosis for the conventional and four improved ACR-TIRADSs are presented in Table 5. ACR-TIRADS had an unnecessary biopsy rate of 62.8% (201/320) and a malignant diagnosis missed rate of 1.1% (8/728). The first three methods significantly reduced the rate of unnecessary biopsies (from 62.8% to 30.0%, 27.1% and 26.8%), but the corresponding missed diagnosis rates were high (from 1.1% to 2.8%, 3.7% and 5.4%, *P* < 0.001). The fourth improved ACR-TIRADS (A or B) had unnecessary biopsy rate of 29.1% and missed diagnosis rate of 1.2% (9/728). The malignant cases missed by the fourth improved ACR-TIRADS are shown in Supplementary Table 7. Among the total of 9 missed cases, 7 were follicular thyroid carcinoma (FTC) and 2 were papillary thyroid carcinoma (PTC). None of these cases exhibited restricted diffusion or reversed halo sign.

**Table 5:** The AUC, unnecessary biopsy rate and malignant missed diagnosis for the conventional and four improved ACR-TIRADSs

Methods	Size (mm)	Benign (n)	Malignant (n)	Total (n)	AUC	*P*	Unnecessary biopsy rate (%)	Malignant missed diagnosis (%)
ACR-TIRADS		201		320	0.887 (0.861–0.909)	< 0.001^*^	62.8	1.1 (8/728)
TI-RADS 3	≥ 25	119	4	123			96.7	
TI-RADS 4	≥ 15	72	29	101			71.3	
TI-RADS 5	≥ 10	10	86	96			10.4	
Restricted diffusion (A)		49		169	0.945 (0.926–0.961)	< 0.001^*^	30.0	2.8 (21/728)
TI-RADS 3	≥ 25	30	5	35			85.7	
TI-RADS 4	≥ 15	9	7	16			56.3	
TI-RADS 5	≥ 10	10	108	118			8.5	
Reversed halo sign (B)		45		166	0.947 (0.928–0.962)	< 0.001^*^	27.1	3.7 (27/728)
TI-RADS 3	≥ 25	33	12	45			73.3	
TI-RADS 4	≥ 15	7	9	16			43.7	
TI-RADS 5	≥ 10	5	100	105			4.8	
A + B		41		153	0.945 (0.926–0.961)	< 0.001^*^	26.8	5.4 (39/728)
TI-RADS 3	≥ 25	33	12	45			73.3	
TI-RADS 4	≥ 15	6	12	18			33.3	
TI-RADS 5	≥ 10	2	88	90			2.2	
A or B		53		182	0.951 (0.932–0.965)	< 0.001^*^	29.1	1.2 (9/728)
TI-RADS 3	≥ 25	30	5	35			19.5	
TI-RADS 4	≥ 15	10	4	14			71.4	
TI-RADS 5	≥ 10	13	120	133			9.7	

*Abbreviations*: *ACR* American Radiology, *TIRADS* Thyroid Imaging Reporting and Data System, *P* the *p* value of Delong test between the AUC of conventional and improved ACR-TIRADSs

## Discussion

In this study, MRI-based morphological features enhanced the diagnostic performance of ACR-TIRADS and considerably decreased the number of nodules categorized as TI-RADS 4 (from 259 to 59, 58, 74, and 43). Among the improved ACR-TIRADSs, the fourth method (A or B) showed the best performance with an AUC of 0.951 compared to 0.887 for conventional ACR-TIRADS (*P* < 0.001). Furthermore, this approach reduced unnecessary biopsy rate to 29.1% without compromising the low malignant missed diagnosis rate (1.2%).

The TI-RADS for thyroid nodules is routinely used for risk stratification and FNA screening of thyroid nodules based on ultrasound characteristics, such as the ACR-TIRADS [11]. A meta-analysis conducted by Castellana M et al. compared the diagnostic performance of different TI-RADS grading system and found that ACR TI-RADS had better performance in selecting thyroid nodules for FNA [24]. Similar findings were reported in other studies [25–28]. However, ACR-TIRADS has a low specificity of 49% at the optimal cutoff of TR4 and requires improvement [12]. In recent years, ultrasound has been utilized to improve the diagnostic accuracy of ACR-TIRADS. The study conducted by Huang et al. [29] revealed a strong correlation between thyroid nodules exhibiting lobulated or irregular borders, punctate echogenic foci, and hypoenhancement on contrast-enhanced ultrasound with malignant tumors. The modified TI-RADS (AUC = 0.863) was considerably superior to ACR-TIRADS (AUC = 0.738) in distinguishing between benign and malignant nodules. Luo et al. [30] employed ultrasound radiomics scoring (Rad-score) in conjunction with ACR-TIRADS to assess its efficacy. While Rad-score demonstrated lesser discriminatory ability than ACR-TIRADS in distinguishing between benign and malignant tumors, the combined approach exhibited superior performance compared to either individual method (AUC: 0.913 vs. 0.899). Notably, no studies investigating the utilization of MRI to improve ACR-TIRADS have been identified.

We have made modifications to the ACR-TIRADS system by integrating restricted diffusion and reversed halo sign on MRI. DWI is a non-invasive imaging modality that captures the microscopic and stochastic motion of water molecules within living tissues, enabling the assessment of diffusion characteristics in vivo. It has been recognized as a valuable imaging biomarker for distinguishing benign and malignant tumors [31, 32]. Previous studies have demonstrated that malignant thyroid nodules exhibited significantly lower ADC values compared to benign nodules, while the optimal threshold for ADC values varies across studies [33–35]. Restricted diffusion, which refers to the presence of areas with high signal on DWI and low signal on ADC maps. This method provides a straightforward and feasible way for evaluating diffusion restriction without relying on specific ADC thresholds. Pathologically, thyroid cancer is characterized by densely packed tumor cells, which can impede the movement of water molecules and manifest as restricted diffusion. In the delay phase, the reversed halo sign presents as a distinctive imaging features characterized by a contrast enhancement that progresses differently in the central and peripheral areas of the lesion. Specifically, the central region of the lesion demonstrates a more rapid clearance of contrast compared to the peripheral region. In our opinion, this imaging pattern likely suggests that the central active proliferation of neoplastic cells results in washout, while the abundant peripheral tumor stroma causes sustained enhancement.

In our analysis of the diagnostic performance of the four improved ACR-TIRADSs with different cutoff values, we observed that most of the improved ACR-TIRADSs exhibited significantly superior diagnostic efficacy compared to ACR-TIRADS, regardless of the size of nodules. This improvement was particularly notable for nodules ranging from 1 to 4 cm in size.

When assessing the diagnostic performance of the four improved ACR-TIRADSs for different pathological nodule types, we observed that the diagnostic efficacy of ACR-TIRADS for malignant nodules was comparable to that of the four improved ACR-TIRADSs, particularly for PTC, which constituted a significant proportion of cases (98.8% VS 94.8%, 95.6%, 91.3%, and 99.2%). The diagnostic efficacy of both the conventional and improved ACR-TIRADSs was relatively low for FTC. Notably, among the improved ACR-TIRADSs, the fourth method (A or B) exhibited the lowest malignant missed diagnosis rate, but even this method failed to accurately diagnose 7 out of the 9 FTC cases. Therefore, poor efficacy in diagnosing benign and malignant follicular thyroid neoplasms (FTNs) is a limitation of the improved ACR-TIRADSs methods. Distinguishing FTC from follicular thyroid adenoma (FTA) relies primarily on postoperative pathological examination to assess capsule and blood vessel invasion which cannot be diagnosed through ultrasound or FNA [36, 37]. Improvements should be investigated for diagnosis of benign and malignant FTN in the future studies. However, for benign nodules, particularly nodular goiter, the improved ACR-TIRADSs consistently demonstrated a substantial enhancement in diagnostic accuracy (39.9% VS 93.9%, 93.9%, 95.2%, and 91.4%). This improvement significantly reduced the need for additional FNA for benign nodules, thereby minimizing the risks associated with invasiveness and potential bleeding, as indicated in the reduction of unnecessary biopsy rate.

There are several shortcomings in this study. Firstly, our study design is retrospective, which inherently introduces selection bias. Cases were selected after surgical treatment, and the exclusion of many benign nodules and nodules chosen for follow-up after fine needle aspiration biopsy would have influenced the results. Secondly, the MRI-based morphological features utilized in this study are somewhat subjective and may vary based on individual interpretation. Lastly, this study was conducted in a single-center setting, and further research involving multiple centers is necessary to validate the effectiveness of MRI-based morphological features in improving the performance of the ACR-TIRADS risk stratification system.

## Conclusion

The study provides evidence that MRI morphological features can help predict malignancy in thyroid nodules and suggests simple methods for combining MRI morphological features with the ACR-TIRADS. The fourth methods improves diagnostic performance reducing the need of biopsy while maintaining a low malignant missed diagnosis rate.

### Supplementary Information

Supplementary Material 1.

## Data Availability

The data sets generated and/or analyzed in the current study were not made public because patients’ personal information was included. Available from the corresponding author upon reasonable request.

## Abbreviations

TI-RADS: Thyroid Imaging Reporting and Data System
ACR: American College of Radiology
MRI: Magnetic resonance imaging
DWI: Diffusion-weighted imaging
CE: Contrast-enhanced
T2WI: T2-weighted imaging
FRFSE: Fast recovery fast spin echo
T1WI: T1-weighted imaging
SE-EPI: Spin-echo echo-planar imaging
FSPGR: Fast spoiled gradient echo
PACS: Picture Archiving and Communication System
ADC: Apparent diffusion coefficient
PPV: Positive predictive value
NPV: Negative predictive value
SD: Standard deviation
ROC: Receiver operating characteristic
AUC: Area under the Receiver operating characteristic curve
CI: Confidence interval
FTC: Follicular thyroid carcinoma
PTC: Papillary thyroid carcinoma
