# Supplementary material for: Thyroid imaging reporting and data system with MRI morphological features for thyroid nodules: diagnostic performance and unnecessary biopsy rate
Source: Cancer Imaging. 2024 Jun 14;24:74. doi: 10.1186/s40644-024-00721-8 (PMC11177423; doi:10.1186/s40644-024-00721-8)
Supplement: Supplementary file 1 — Supplementary Material 1. [file 40644_2024_721_MOESM1_ESM.docx]

**Supplementary Material**

**Table 1.** Parameters of MRI Sequence

|  | Plane | TI-RADS | TE | Slice thickness | Gap between slices | NEX | FOV | MaTI-RADSix size | Sequence |
| --- | --- | --- | --- | --- | --- | --- | --- | --- | --- |
| T2WI with fat suppression | coronal | 1300 | 81.2 | 4 | 0.5 | 4 | 20 | 288×192 | FRFSE |
| T1WI | axial | 540 | 10.6 | 4 | 0.5 | 2 | 20 | 288×224 | FSE-XL |
| T2WI with fat suppression | axial | 3000 | 86.9 | 4 | 0.5 | 2 | 20 | 320×224 | FRFSE |
| DWI | axial | 6550 | 85 | 4 | 0.5 | 8 | 20 | 128×128 | SE-EPI |
| ConTI-RADSast-enhanced T1WI | axial | 5.5 | 1.6 | 4 | -2 | 1 | 25 | 256×192 | FSPGR |
| Units |  | ms | ms | mm | mm |  | cm |  |  |

Abbreviations: TI-RADS, repetition time; TE, echo time; NEX, number of excitations; FOV, field of view; T2WI, T2-weighted imaging; T1WI, T1-weighted imaging; DWI, diffusion-weighted imaging; FRFSE, fast recovery fast spin echo; FSE, fast spin echo; SS-EPI, single shot echo-planar imaging; FSPGR, fast spoiled gradient echo.

**Table 2**. Five US features in ACR-TIRADS

| Score | Composition | Echogenicity | Margin | Shape | Calcification |
| --- | --- | --- | --- | --- | --- |
| 0 | Cystic or almost cystic or spongy | No echogenicity | Smooth or unclear | Horizontal | No or with comet tail sign |
| 1 | Cystic solid | Hyperechoic or isoechoic | - | - | Coarse calcification |
| 2 | Solid or almost completely solid | Hypoechoic | Irregular or lobulated | - | Peripheral calcification |
| 3 | - | Very hypoechoic | ExTI-RADSa-thyroidal invasion | Vertical | Microcalcification |

Abbreviations: ACR, American College of Radiology; TI-RADS, Thyroid Imaging Reporting and Data System; US, UlTI-RADSasonography.

**Table 3.** ACR-TIRADS and management comments

| Classification | US score | Malignancy risk rate(%) | Recommended FNA cut-off value |
| --- | --- | --- | --- |
| Benign | 0 point | < 2 | Not required |
| No suspicion | 2 points | < 2 | Not required |
| Mildly suspicious | 3 points | 5 | ≥ 2.5 cm |
| Moderately suspicious | 4-6 points | 5-20 | ≥ 1.5 cm |
| Highly suspicious | ≥7 points | ≥ 20 | ≥ 1 cm |

Abbreviations: ACR, American College of Radiology; TI-RADS, Thyroid Imaging Reporting and Data System; US, UlTI-RADSasonography.

**Table 4.** Comparison of the upgraded and downgraded nodules in different improved methods

| Methods | Upgraded (n) | Downgraded (n) |
| --- | --- | --- |
| Restricted diffusion (A) | 475 | 253 |
| Reversed halo sign (B) | 491 | 237 |
| A + B | 189 | 539 |
| A or B | 427 | 301 |

Data are expressed as the number of nodules.

**Table 5.** Comparison of conventional and four improved ACR-TIRADSs across three different groups based on thyroid nodule size

| Methods and Diagnosis | | <1cm (n=240) | | 1-4cm (n=398) | | >4cm (n=90) | |
| --- | --- | --- | --- | --- | --- | --- | --- |
|  |  | Malignant | Benign | Malignant | Benign | Malignant | Benign |
| Restricted diffusion | Present | 103 | 13 | 111 | 10 | 12 | 4 |
|  | Absent | 21 | 103 | 23 | 254 | 5 | 69 |
|  | *p* value | <0.001* | | <0.001* | | <0.001* | |
|  | Accuracy (%) | 83.1 | 88.8 | 82.8 | 96.2 | 70.6 | 94.5 |
| Reversed halo sign | Present | 104 | 10 | 111 | 9 | 3 | 0 |
|  | Absent | 20 | 106 | 23 | 253 | 14 | 73 |
|  | *p* value | <0.001* | | <0.001* | | 0.006 | |
|  | Accuracy (%) | 83.9 | 91.4 | 82.8 | 96.6 | 17.6 | 100.0 |
| ACR-TIRADS (≥4) | Malignant | 120 | 70 | 134 | 106 | 13 | 19 |
|  | Benign | 4 | 46 | 0 | 158 | 4 | 54 |
|  | *p* value | <0.001* | | <0.001* | | <0.001* | |
|  | Accuracy (%) | 69.2 (166/240) | | 73.4 (292/398) | | 74.4(67/90) | |
| Restricted diffusion (A) (≥4) | Malignant | 118 | 22 | 125 | 18 | 12 | 4 |
|  | Benign | 6 | 94 | 9 | 246 | 5 | 69 |
|  | *p* value | <0.001* | | <0.001* | | <0.001* | |
|  | Accuracy (%) | 88.4 (212/240) | | 93.2 (371/398) | | 90.0 (81/90) | |
| Reversed halo sign (B) (≥4) | Malignant | 118 | 22 | 125 | 16 | 5 | 0 |
|  | Benign | 6 | 94 | 9 | 248 | 12 | 73 |
|  | *p* value | <0.001* | | <0.001* | | <0.001* | |
|  | Accuracy (%) | 88.4 (212/240) | | 93.7 (373/398) | | 86.7 (78/90) | |
| A+B (≥4) | Malignant | 111 | 15 | 120 | 11 | 5 | 0 |
|  | Benign | 13 | 101 | 14 | 253 | 12 | 73 |
|  | *p* value | <0.001* | | <0.001* | | <0.001* | |
|  | Accuracy (%) | 88.4 (212/240) | | 93.7 (373/398) | | 86.7 (78/90) | |
| A or B (≥4) | Malignant | 124 | 33 | 130 | 23 | 12 | 4 |
|  | Benign | 0 | 83 | 4 | 241 | 5 | 69 |
|  | *p* value | <0.001* | | <0.001* | | <0.001* | |
|  | Accuracy (%) | 86.3 (207/240) | | 93.2 (371/398) | | 90.0 (81/90) | |

Data are expressed as the number of nodules, with percentages in parentheses. * *p*<0.05.

**Abbreviations:** ACR, American Radiology; TIRADS, Thyroid Imaging Reporting and Data System.

**Table 6.** Comparison of different MRI-based morphological features and ACR-TIRADSs across different pathological types

| Methods and Diagnosis | | PTC  (n=252) | FTC  (n=15) | MTC  (n=4) | UTC  (n=2) | NG  (n=313) | Adenoma  (n=43) | AG  (n=61) | NHT  (n=23) | Subacute thyroiditis  (n=11) |
| --- | --- | --- | --- | --- | --- | --- | --- | --- | --- | --- |
| Restricted diffusion | Present | 211 | 8 | 4 | 2 | 5 | 8 | 3 | 2 | 9 |
|  | Absent | 41 | 7 | 0 | 0 | 308 | 35 | 58 | 21 | 2 |
|  | Accuracy (%) | 83.7 | 53.3 | 100.0 | 100.0 | 98.4 | 81.4 | 95.1 | 91.3 | 18.2 |
| Reversed halo sign | Present | 214 | 0 | 2 | 1 | 11 | 39 | 2 | 1 | 1 |
|  | Absent | 38 | 15 | 2 | 1 | 302 | 4 | 59 | 22 | 10 |
|  | Accuracy (%) | 84.9 | 0.0 | 50.0 | 50.0 | 96.5 | 90.7 | 96.7 | 95.7 | 90.9 |
| ACR-TIRADS (≥4) | Malignant | 249 | 4 | 4 | 2 | 125 | 20 | 27 | 14 | 9 |
|  | Benign | 3 | 11 | 0 | 0 | 188 | 23 | 34 | 9 | 2 |
|  | Accuracy (%) | 98.8 | 26.7 | 100.0 | 100.0 | 39.9 | 53.5 | 55.7 | 39.1 | 18.2 |
| Restricted diffusion (A) (≥4) | Malignant | 239 | 8 | 4 | 2 | 19 | 9 | 7 | 4 | 2 |
|  | Benign | 13 | 7 | 0 | 0 | 294 | 34 | 54 | 19 | 9 |
|  | Accuracy (%) | 94.8 | 53.3 | 100.0 | 100.0 | 93.9 | 79.2 | 88.5 | 82.6 | 18.2 |
| Reversed halo sign (B) (≥4) | Malignant | 241 | 0 | 4 | 1 | 19 | 5 | 6 | 2 | 2 |
|  | Benign | 11 | 15 | 0 | 1 | 294 | 38 | 55 | 21 | 9 |
|  | Accuracy (%) | 95.6 | 0.0 | 100.0 | 50.0 | 93.9 | 88.4 | 90.2 | 91.3 | 81.8 |
| A + B (≥4) | Malignant | 230 | 0 | 4 | 1 | 15 | 2 | 5 | 2 | 2 |
|  | Benign | 22 | 15 | 0 | 1 | 298 | 41 | 56 | 21 | 9 |
|  | Accuracy (%) | 91.3 | 0.0 | 100.0 | 50.0 | 95.2 | 95.3 | 91.8 | 91.3 | 81.8 |
| A or B (≥4) | Malignant | 250 | 8 | 4 | 2 | 27 | 12 | 8 | 4 | 9 |
|  | Benign | 2 | 7 | 0 | 0 | 286 | 31 | 53 | 19 | 2 |
|  | Accuracy (%) | 99.2 | 53.3 | 100.0 | 100.0 | 91.4 | 72.1 | 86.9 | 82.6 | 18.2 |

Data are expressed as the number of nodules unless otherwise indicated.

**Abbreviations:** PTC, papillary thyroid carcinoma; FTC, follicular thyroid carcinoma; MTC, medullary thyroid carcinoma; UTC, undifferentiated thyroid carcinoma; NG, nodular goiter; AG, adenomatous goiter; NHT, nodular hashimoto thyroiditis; ACR, American College of Radiology; TIRADS, Thyroid Imaging Reporting and Data System.

**Table 7.** Malignant missed diagnosis cases in the fourth method (ResTI-RADSicted diffusion or Reversed halo sign)

| Age | ACR-TIRADS | The fourth method (A or B) | ResTI-RADSicted diffusion (A) | Reversed halo sign (B) | Pathology type |
| --- | --- | --- | --- | --- | --- |
| 27 | TI-RADS 3 | TI-RADS 2 | Absent | Absent | FTC |
| 36 | TI-RADS 4 | TI-RADS 3 | Absent | Absent | FTC |
| 34 | TI-RADS 4 | TI-RADS 3 | Absent | Absent | PTC |
| 45 | TI-RADS 4 | TI-RADS 3 | Absent | Absent | FTC |
| 35 | TI-RADS 4 | TI-RADS 3 | Absent | Absent | PTC |
| 50 | TI-RADS 4 | TI-RADS 3 | Absent | Absent | FTC |
| 43 | TI-RADS 4 | TI-RADS 3 | Absent | Absent | FTC |
| 47 | TI-RADS 4 | TI-RADS 3 | Absent | Absent | FTC |
| 65 | TI-RADS 3 | TI-RADS 2 | Absent | Absent | FTC |

**Abbreviations:** ACR, American Radiology; TIRADS, Thyroid Imaging Reporting and Data System; FTC, Follicular thyroid carcinoma; PTC, Papillary thyroid carcinoma.
